# Hospital Meetings

**Published:** 1901-09-14

**Authors:** 


### HOSPITAL MEETINGS.

### LONDON HOSPITAL.

At the quarterly special general court of the London Hospital, on Wednesday, the 4th inst., Mr. J. H. Buxton, the Treasurer, took the chair.

The Secretary and House Governor, Mr. G. Q. Roberts (the minutes of the last meeting having been read and confirmed), read the quarterly report. The house committee, while prefacing that the governors were aware of the enormous amount of building work which is being busily carried forward, stated that they were pleased to be able to report that much of it is nearly completed. Notwithstanding all this work going on, the figures relating to patients treated show that the numbers have been higher than ever. The fact cannot be emphasised too much that building operations involving an expenditure of over £370,000 are being carried on so far without the closing of a single bed or diminishing in any degree the work in the treatment of cases. The pressure is felt most severely in the out-patient department, and this department has become a very serious problem. The completion of the new buildings in Turner Street is being pushed forward so that it may be possible to use at any rate the ground floor in the early part of next year, leaving the special departments to be finished later on. Nothing can be done with the rearrangement of the west wing until the new out-patient department is finished, but the east wing can be got forward with, and definite instructions have been given to the architect as to the work to be proceeded with. It is proposed to put the kitchen on the roof of the east wing, and to set aside the site of the present laundry for the accommodation of the porters. The purchase has been completed, for a sum of £6,407, of the John Baker's Almshouses, and plans have been prepared and instructions have been given for the building of a new laundry there as soon as possible. Some of the governors, the report observed, might have had trouble in getting to the court-room that day, having had to pass through the receiving-room, and this is owing to the fact that the greater part of the main corridor is blocked. This will give some idea of the difficulties experienced in carrying on the work at present. Mosaic is being laid down in this corridor, and the walls are being lined with opalite and the ceilings with emdeca. There is no doubt that the hospital will be immensely improved, for on completion there will then be the very best method available of keeping the main entrance and ground-floor corridor clean and in good order. Part of the new receiving-room is in use, and the old part is being rearranged with a view to bringing the whole department up to what are considered to be modern needs. The new store-rooms have been finished and are in occupation with most satisfactory results. The isolation block is finished, and arrangements have been made for electric light throughout, and the whole will be ready for occupation within a week or two. General cases will be put in there in the first instance whilst the rebuilding of the east wing is going on. This is quite a model block, and one which, when finished, will be of the utmost value, and, as previously announced, it has been given to the hospital by an anonymous donor at a cost of £32,000. A great number of nurses are still being housed in Newark Street, Turner Street, and Philpot Street, the accommodation for them being lamentably short. It is intended that this matter shall be dealt with at an early date, and in a thorough and comprehensive manner. On the resignation of Dr. Hewitt the whole department connected with the administration of anæsthetics in the hospital was carefully considered by the committee, and Dr. Probyn-Williams succeeded Dr. Hewitt as senior instructor in anæsthetics. Two assistants have been appointed, Dr. H. Hilliard and Dr. E. W. Clapham, so that on every afternoon when important operations are going on in the theatre a responsible anæsthetist is present. The pathological department of the hospital, for which the new building was reported as finished at the last Court, was opened by Sir Henry Roscoe on July 10. Lord Lister and Professor Clifford Allbutt both spoke in the highest terms of the excellent work done by the Governors in starting such work in the East End of London, where the enormous clinical material could be made use of for the advancement of general medical knowledge. It can be said proudly that no better department than this exists anywhere. Dr. Bulloch has been appointed chief of this department, and Dr. Wm. Hunter as his assistant. The Finsen Light treatment for lupus, which was introduced at this hospital by Queen Alexandra, continues to be a great success. The lamps which were originally installed have been of the utmost service, but it has been discovered that by a small and cheaper lamp equally effective work may be done. In the 15 months since the light was installed, 213 patients have been treated. Dr. Andrews has been re-elected obstetric registrar for nine months from July 31st, and leave has been given to Mr. Newland to resign his position as surgical registrar to take up a practice abroad. A gentleman will be appointed to succeed him on the 1st prox. The committee allude with deep regret to the death of their colleague, Sir Cuthbert Peek; Sir Cuthbert, they observe, having been one of the kindest and most generous of men. To fill the vacancy caused by his death, the committee unanimously recommended the election of Mr. Percy Tarbutt as a member of the house committee. Mr. Tarbutt has shown great interest in the hospital, and has collected a sum of no less than £7,500 for the purpose of endowing a lupus lamp. Once more the committee particularly call attention to the "forget-me-not" bond which has been instituted as a means of affording people who are not able to contribute on a large scale the means of giving something to the hospital. If 50,000 of these books could be issued it would mean £10,000 a year to the hospital; 14,000 have been issued in the first year. The present year's working is considered by the committee to be satisfactory, and in recognition of their services to the fund, they recommended the election of the following gentlemen as Life Governors of the hospital:—Mr. Alfred James Sheffield, Mr. Thomas Edward Grigsby, Mr. Thomas Hughes. The following donations have been received:—£1,000 each—Mr. Henry Bonas, the Worshipful Company of Grocers, and Mr. Peregrine Purvis; £500 each—Mr. N. Matheson McWharrie and C. G. T.; £250 each—Chapel offertory, Anon., and Mrs. Bingen; £112—the Rev. Richard Free; £100 each—Hon. Mrs. Alan Johnstone, Miss C. Lawrence, and Miss E. M. Lawrence. The following legacies have also been received:—£1,025 from David Hughes Trust; £500 each—R. Bowerman West, Miss Eliz. Ainslie, and H. C. Knowles; £100 each—D. Benito y Jimenez, Edw. Knightley, and H. W. Segelke.

Mr. Buxton moved the adoption of the report, and Sir

Edmund Hay Currie (one of the vice-presidents and chairman of the Hospital from 1868 to 1877) seconded the motion in a few graceful words of compliment to the present management and confidence that, great as is the work being done, a greater can be done in the future. The motion having been carried unanimously, formal votes of approval of the election of the proposed new life-governors, etc., were put and agreed to.

The proceedings then terminated.

